# *Kaniuwatewara* (when we get sick): understanding health-seeking behaviours among the Shawi of the Peruvian Amazon

**DOI:** 10.1186/s12889-021-11574-2

**Published:** 2021-08-16

**Authors:** Alejandra Bussalleu, Pedro Pizango, Nia King, James Ford, I. H. A. C. C. Research Team, Sherilee L. Harper

**Affiliations:** 1 Faculty of Public Health and Administration, Universidad Peruana Cayetano Heredia, Honorio Delgado, 430 Lima, Peru; 2 Communidad Nativa Balsapuerto, Alto Amazonas, Communidad Nativa, Loreto Peru; 3 Department of Population Medicine, University of Guelph, 50 Stone Road East, Guelph, Ontario Canada; 4 Department of Medicine, Queen’s University, 99 University Avenue, Kingston, Ontario Canada; 5 Priestly International Centre for Climate, University of Leeds, Leeds, UK; 6 Indigenous Health Adaptation to Climate Change Research Team: Lea Berrang-Ford, Cesar Carcamo, Patricia Garcia, Shuaib Lwasa, Didacus B. Namanya, Edmonton, Canada; 7 School of Public Health, University of Alberta, 116 St & 85 Ave, Edmonton, Alberta Canada

**Keywords:** Peru, Health-seeking behaviour, Healthcare access, Indigenous, Shawi, Intercultural health, Intercultural healthcare

## Abstract

**Background:**

Detailed qualitative information regarding Indigenous populations’ health-seeking behaviours within Peru’s plural healthcare system is lacking. Such context-specific information is prerequisite to developing evidence-based health policies and programs intended to improve health outcomes for Indigenous populations. To this end, this study aimed to characterize health-seeking behaviours, factors affecting health-seeking behaviours, and barriers to obtaining healthcare in two Indigenous Shawi communities in Peru.

**Methods:**

Community-based approaches guided this work, and included 40 semi-structured interviews and a series of informal interviews. Data were analysed thematically, using a constant comparative method; result authenticity and validity were ensured via team debriefing, member checking, and community validation.

**Results:**

Shawi health-seeking behaviours were plural, dynamic, and informed by several factors, including illness type, perceived aetiology, perceived severity, and treatment characteristics. Traditional remedies were preferred over professional biomedical healthcare; however, the two systems were viewed as complementary, and professional biomedical healthcare was sought for illnesses for which no traditional remedies existed. Barriers impeding healthcare use included distance to healthcare facilities, costs, language barriers, and cultural insensitivity amongst professional biomedical practitioners. Nevertheless, these barriers were considered within a complex decision-making process, and could be overridden by certain factors including perceived quality or effectiveness of care.

**Conclusions:**

These findings emphasize the importance of acknowledging and considering Indigenous culture and beliefs, as well as the existing traditional medical system, within the professional healthcare system. Cultural competency training and formally integrating traditional healthcare into the official healthcare system are promising strategies to increase healthcare service use, and therefore health outcomes.

**Supplementary Information:**

The online version contains supplementary material available at 10.1186/s12889-021-11574-2.

## Background

Globally, health indicator data for Indigenous populations reveal striking disparities with respect to morbidity and mortality when compared to their non-Indigenous counterparts [1–4]. These disparities reflect several inequities with respect to social determinants of health and healthcare access, as well as histories of colonization and marginalization [1, 2].

Shawi, also known as Chayahuita or *Kampo piyapi*, are an Indigenous group that reside largely in isolated riverine communities in north-eastern Peru. Few (12.9%) Shawi communities have governmental primary healthcare posts, and less than half (37.9%) have first-aid kits managed by a community-designated health promoter trained to help during basic health emergencies; however, these proportions are among the highest for Amazonian Indigenous groups [5]. Shawi health indicators remain low: the infant mortality rate of 125 deaths per 1000 live births is among the highest of the Loreto region, and malaria, leishmaniasis, and other infectious diseases remain problematic [6, 7].

Although the Peruvian Ministry of Health has worked to improve disadvantaged populations’ health, including establishing a national health insurance program (Seguro Integral de Salud [SIS]) for children, pregnant women, and impoverished adults, health inequities persist [2, 8, 9]. Furthermore, even in communities where physical access to biomedical healthcare has improved, healthcare utilization and health outcomes did not necessarily improve [2, 10]. To this end, effectively improving health outcomes in remote Amazonian Indigenous communities requires an understanding of how these populations navigate Peru’s plural healthcare system [11].

Health-seeking behaviour (HSB) refers to the actions taken by an individual who, under his or her own perception, is ill or in need of care, in order to find a suitable remedy or treatment [12]. A variety of structural, socioeconomic, cultural, psychological, and demographic factors can influence HSBs, however the specific factors, and the relative importance of each, vary between populations [8, 9, 11, 13, 14]. Although a small number of quantitative studies have investigated HSBs in remote Peruvian Amazonian communities [8, 9, 15], detailed qualitative information regarding the reasonings behind HSBs is lacking [13]. Such context-specific information is necessary to develop evidence-based health policies and programs intended to enhance healthcare access and improve health outcomes [11, 16, 17].

To this end, in order to inform future health policies and programs, this research examined HSBs amongst two Indigenous Shawi communities. The aim of this study was to characterize the plural healthcare system, common health-seeking pathways, factors affecting HSBs, and barriers to obtaining healthcare.

## Methods

This study was conducted as a project under the Indigenous Health Adaptation to Climate Change (IHACC) program, a multi-year, community-based initiative working with Indigenous populations in Peru, Canada, and Uganda to understand climate change impacts on health. This study built upon preliminary community-based research efforts, during which community members identified understanding health-seeking behaviours as a priority research area.

A combination of qualitative methods was employed to develop an understanding of the patterns of decisions and actions involved in local HSBs [12, 13]. As outlined in Bussalleu et al. (2021), a community-based participatory research approach was used [18–21]. Through the use of frequent community assemblies and informal conversations, community members helped guide all stages of the research process, including developing the research questions and semi-structured interview guide, participating in data collection, and analyzing the research results. Member-checking was also completed with community members to verify preliminary findings and to allow community members to provide additional feedback. Final research results were disseminated through community assemblies.

### Location

**Fig. 1:**
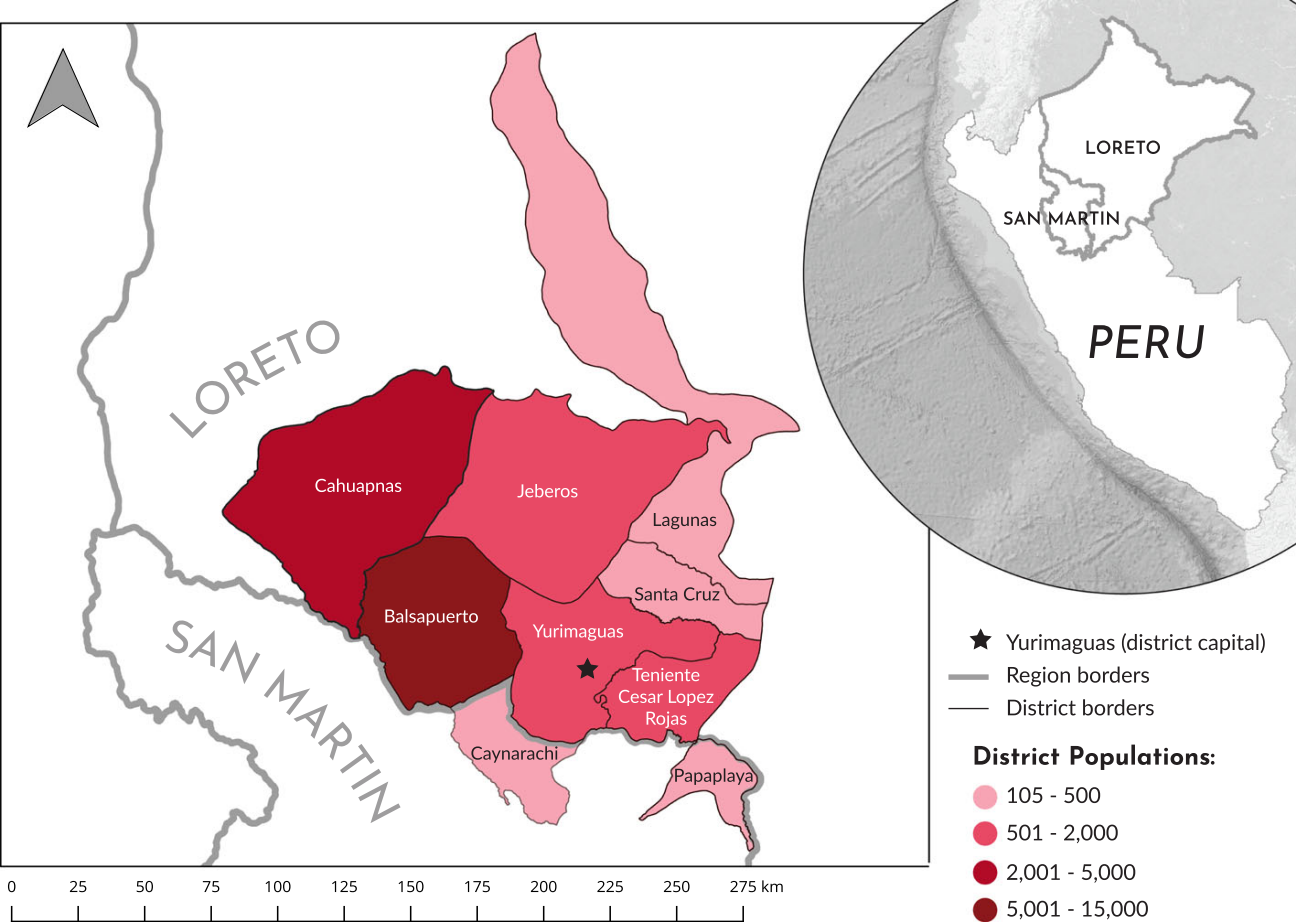
Peruvian districts where Shawi people reside. The two communities are not specified for confidentiality reasons. Figure created by Alexandra Sawatzky

Two Shawi communities (Communities A and B), located along a river in the Balsapuerto district of Loreto region, participated in this study (Fig. 1). To respect the confidentiality of respondents, we do not specify the name of the river or specific communities. Both communities lack water and sanitation infrastructure and are a several hour drive from the nearest hospital in the provincial capital, Yurimaguas (Table 1). Subsistence agriculture, gathering, and fishing form the basis of household livelihoods, and traditional customs and beliefs are widely held in these communities.

**Table 1:** Community characteristics of two Shawi communities, Peru, in 2014

	Community A	Community B
Population	278	570
Total # of households	36	64
% of households interviewed	31	34
Rural satellite phone	Yes	Yes
Water and sanitation services	None	None
Local health facilities	None; 1 h by foot to nearest health post	Health post with 2 nurse technicians
Travel time to nearest hospital	~ 2 h drive	~ 2 h drive + several hour walk

### Data collection

Community-wide engagement and oral informed consent were acquired from community leaders before the study commenced. Prior to household data collection, the voluntary nature of the study was explained to participants; participants then either provided written consent, or oral consent accompanied by a fingerprint. If participants consented, the interview was audio-recorded; if not, detailed research notes were taken throughout the interview. The study was approved by the Institutional Ethics Committee at Universidad Peruana Cayetano Heredia.

#### Participants

Participant sampling is outlined in Bussalleu et al. (2021) [21]. Interviews were conducted at the household-level because preliminary data collection suggested that family consultations highly influenced HSBs. All households that were invited to take part in the study participated.

Interviewed households in both communities had an average of 4 children. The age of interviewees ranged from 17 to 64, but was an average of 42 in Community A and 38 in Community B. The highest level of education attained by female and male interviewees was sixth and eleventh grade, respectively. However, most female participants had no formal education.

#### Semi-structured interviews

In depth, semi-structured interviews were conducted with 33 households (A: *n* = 11; B: *n* = 22), four representatives from the professional healthcare sector, and three representatives from the traditional healthcare sector. The interviews explored available healthcare options, common HSBs, and factors affecting HSBs. A HSB pathway model was used to represent HSB as a dynamic process [11]. The open-ended interview guide was developed for this study (see Additional File 1) and was validated during pilot data collection with the local Indigenous researcher, community members, and bilingual Shawi teachers. Additional information on semi-structured interviews can be found in Bussalleu et al. (2021) [21]. To crosscheck information and interpretation, some questions were asked twice and some households were interviewed in multiple seasons.

#### Informal interviews

As outlined in Bussalleu et al. (2021), informal interviews in the form of unstructured individual or group conversations were also conducted with community members, herbalists, healers, and governmental healthcare workers, which provided insights that further contextualized findings. A female interpreter assisted the researcher when speaking with women alone, which allowed participants to speak more openly about pregnancies, contraceptives, and general female HSBs. Based on previous research conducted in these communities, which characterized community members’ preference for an iterative approach to informed consent, consent was obtained from each participant, and was continually revisited throughout the project – from the identification of the research question in collaboration with the community, to the research results dissemination [22].

#### Cultural immersion

The first author lived in the communities for over 1 year, which facilitated cultural immersion and provided further in-depth understanding and contextualization of HSBs. During her time in the communities, the researcher worked closely with local herbalists, as well as the local researcher and his family, who all provided important insights into Shawi culture and medical systems. By engaging in daily activities, the researcher became well integrated into the community and was better able to understand community behavioural patterns [23, 24]. It also ensured that community members felt comfortable around her, thus maximizing data accuracy [23]. Throughout their time in the community, the research team used a journal to document subjective experiences and reflections; a separate journal was used to document objective observations.

### Data analysis

The constant comparative data analysis approach, which made use of an inductive codebook, is described in detail by Bussalleu et al. (2021) [21]. Community members were given the opportunity to validate and clarify preliminary findings during community assemblies and individual meetings. Stata (Statacorp™ v10) was used to calculate percentages and frequency counts for specific information (e.g. HSB strategies).

### Study strengths and limitations

Data reliability was maximized using a variety of strategies. Firstly, triangulation, where a combination of complementary data sources are used, was employed [24, 25]. Secondly, as described above, member checking allowed community members to verify findings and provide feedback [19, 24, 25]. Lastly, observations were also discussed with other researchers who had previously lived in the communities to ensure data reliability.

Nevertheless, the challenges associated with qualitative cross-language research can influence a study’s accuracy [26]. In an attempt to mitigate these challenges, a local Indigenous researcher with a diploma in intercultural health, who is an experienced research translator, was recruited [24]. This study was conducted in two Shawi communities that may differ from other communities and ethnic groups in many ways, including geography and access to healthcare services. These results may therefore not be generalizable to other Indigenous communities.

## Results

### Available health-seeking behaviour options

Households reported using various and multiple approaches to address their health concerns. The four major HSBs were: visiting a governmental health post; self-treating with pharmaceuticals; self-treating with traditional home remedies; and visiting a traditional health practitioner. These were categorized into three sectors: the professional sector which includes governmental healthcare; the popular sector which includes both forms of self-treatment; and the traditional sector which includes traditional health practitioners [27–29]. To determine the most appropriate HSB for a particular illness, participants reported that they first consulted their spouse, and occasionally sought subsequent advice from other family members. In the case of a child, mothers reportedly recognized the illness first, and a joint treatment decision was made with their spouse.

#### Governmental health facilities

Almost all residents of both communities were part of the SIS program, and could therefore receive subsidized or free treatment in the governmental facility assigned to their community. For primary healthcare, residents of Community A must travel 1 h by foot, while Community B has its own health post. Secondary care for both communities was located in Balsapuerto, which takes between seven and 8 h on foot or by boat from each community. Tertiary care was located in Yurimaguas.

Participants described that men play an important role when seeking governmental healthcare, as they are responsible for arranging transportation, paying for treatment and other expenses (e.g. accommodation when seeking care in the city), and, due to their comparably higher Spanish proficiency, conversing with government healthcare providers.

#### Self-treatment with pharmaceuticals purchased in the city

Participants reported that the household head purchased pharmaceuticals from *bodegas* (small grocery stores) or pharmacies in Yurimaguas. Commonly purchased pharmaceuticals included paracetamol, ibuprofen, aspirin, and vitamins, which are sold without prescription. Two nurse technicians expressed their concerns regarding product quality from *bodegas*, arguing that these pharmaceuticals could cause allergic reactions or physical discomfort, which could lead to rejection or fear of pharmaceuticals, and consequently affect future treatment choices.

#### Self-treatment with traditional home remedies

Members of both communities widely used traditional remedies for illness treatment and prevention. Knowledge of traditional remedies was transmitted within families. As one participant explained, “*I learned from my mother. I used to watch her preparing the plants and now that I have my own children I do the same things she did”* (Woman, 20–30 years old, Community B).

With the exception of families containing a male traditional healer or herbalist, women were primarily responsible for planting and maintaining medicinal plants, as well as making traditional remedies.

#### Traditional health practitioners

Participants classified traditional health practitioners into three groups: *vegetalistas* (herbalists), *curanderos* or *brujos* (healers), and *penunturu’sa* (shamans).

Participants described that herbalists can recognize and use dozens of medicinal plants for remedies. This knowledge could be obtained from several sources including attending government courses on phytotherapeutic remedies that were offered from 1990 to 2001, observing other herbalists, or asking spirits who manifest their teaching through dreams. As one traditional health practitioner explained,

*When my children are sick, I ask the spirits to help me. In my sleep they show me what plants to use, how to prepare them, and where to get them. The next day, I go to the* monte [woods] *and I find the plants they showed. I remember [the spirits´] instructions and test the remedies with my kids. Then people ask for my help.* Herbalist, Community B

Some herbalists reported including pharmaceuticals in their preparations, including mixing coconut water with Sal de Andrews to treat fever, and paracetamol to treat headaches.

In contrast to herbalists, healers reportedly relied on a combination of traditional remedies and alternative methods for treatment. Healers were viewed as experts in restoring energy imbalances and extracting dark energies from people’s bodies. Tobacco is the preferred tool used during healing sessions to purify the surroundings, provide protection, and/or extract unwanted energies from an affected person. Along with the *ícaros* (songs performed during healing rituals), healers smoked the tobacco on the patient’s energetic points (temple, forehead, heart, back, and hands) or where the illness was located, to alleviate pain and heal the body and spirit.

Shamans were also considered healers, but, as distinguished by participants, have stronger powers and can use spirits’ powers to heal others. Participants described that shamans diagnose and treat illnesses using a variety of plants, including the *Ka’pi’* or *Ayahuasca* (*Banisteropsis caapi*). Participants explained that shamans learn through visions and dreams that the initiates experience while dieting; success in healing and the level of power and knowledge acquired was reported to depend on the length of the diets and the number of plants ingested. Few community members become shamans due to the extensive commitment and sacrifice required. As described by participants, in the previous years, 14 Shawi shamans were killed around Balsapuerto, allegedly for sorcery, political, religious, and racist reasons, and as punishment for the district’s high infant mortality rates [30]. The low availability of shamans was partly attributed to these incidents, since shamans were reportedly hiding their powers for fear of being mistaken for sorcerers, who have the power to make people sick. Shamans were located in a variety of communities, ranging from one to 8 days away.

When visiting local traditional practitioners, female participants described that, although not mandatory, the presence of their husband was preferable. When seeking treatment from a shaman in an adjacent community, spouses often travelled together, as the male was responsible for payment.

### Health-seeking behaviour pathways

**Fig. 2:**
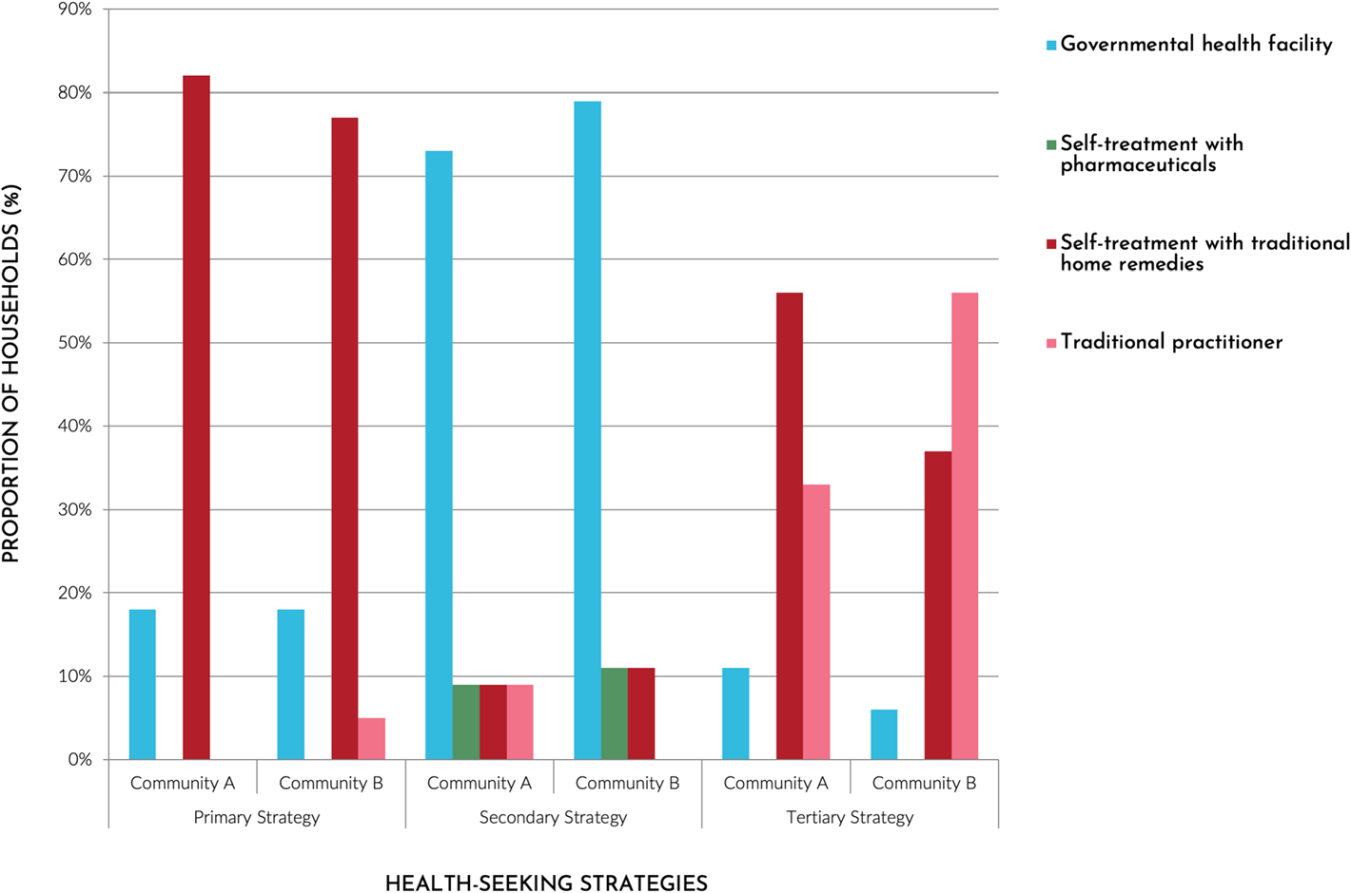
Primary, secondary, and tertiary health-seeking strategies for common illnesses in two Shawi communities, Peru

Participants were asked to provide a detailed description of their HSBs for ‘common’ ailments (e.g. diarrhoea, *calentura* (increased body heat)). For such illnesses, more than three quarters (A: 82%; B: 77%) of households’ primary HSB was self-treatment with traditional home remedies (Fig. 2). As one mother described,

*When my children get sick I always give them plants. I use* malva *for fever.* [ … ] *Now we have longer summers and children get overheated. I wet their heads with* malva chapeada *and in one night they feel better.* Woman, 30-40 years old, Community A

Conversely 18% of households in each community reported using governmental healthcare as their primary HSB due to their limited medicinal plant knowledge.

If the first strategy was deemed unsuccessful, approximately three quarters (A: 73%; B: 79%) of households reported subsequently visiting a governmental health facility. Participants explained that if medicinal plants were not curing the ailment, then pharmaceuticals from the health post were trusted.

As a tertiary HSB, 56% of households in Community A returned to self-treatment with traditional home remedies, compared to 31% of households in Community B. In contrast, more than half (56%) of Community B households consulted a traditional practitioner as a tertiary HSB, compared to one third (33%) of Community A households.

When considering all stages of HSBs, only one household in Community A used neither traditional home remedies nor a traditional practitioner due to not believing in their efficacy. Only one household in Community A reported never having used pharmaceuticals. It is noteworthy that although all households reported several HSBs, almost all households cited using treatments sequentially rather than in parallel.

### Factors influencing health-seeking behaviours

Households’ decisions regarding primary HSB were based on a variety of factors, including: illness type, perceived aetiology, perceived severity, and treatment characteristics.

#### Illness type

Local concepts of illness type were a major determinant of primary HSBs. Traditional home remedies were considered the main option for preventing and treating everyday maladies. Visiting a governmental health facility was also considered effective for everyday illnesses; however, participants preferred using medicinal plants when possible. Healers were sought for *unknown illnesses,* which caused unbearable pain, and for treating children affected by *susto* (acute fear) and *cutipados* (spirits of the forest). In contrast, shamans were considered to be the only effective treatment option for *serious unknown illnesses,* or other traditional illnesses caused by spirits or sorcery, including *daño* (harm caused by sorcery). It is important to note, however, that participants described that good traditional practitioners would acknowledge the limitations of traditional medicine and recommend what is best for the patient. As one man explained, “*Sometimes I consult [community’s healer] and if he knows he cannot cure me, then he tells me that I should go to the health post.*” (Man, 40–50 years old, Community A). As such, although participants may initially seek a healer, they may be referred to a governmental health post.

Governmental healthcare was perceived to be most effective for treating ‘new diseases’, physical ailments, and pain. ‘New diseases’ included AIDS, yellow fever, cancer, cholera, and the flu, for which no traditional remedies exist. Although participants mentioned the existence of traditional remedies for malaria, most acknowledged that pharmaceuticals were more effective. Governmental healthcare was also often sought for pregnancy check-ups, childbirth, contraceptives, and monthly child check-ups. However, according to a health technician, this was typically only to collect JUNTOS money, a national cash transfer program that pays families if mothers have regular pregnancy appointments and children have regular health check-ups and attend school.

#### Perceived illness aetiology

Local concepts of illness aetiology contributed to HSBs due to the differing perceived effectiveness of treatments for particular causal factors. This was best exemplified by the differential HSBs for childhood diarrhoea between the two communities. In Community A, households reported that ingesting microbes from contaminated river water caused the majority of diarrhoea cases, and that this was best treated with pharmaceuticals from the health post. This knowledge reportedly originated from radio advertisements or from the community’s herbalist who had been taught by government staff. In contrast, in Community B, diarrhoea was ascribed to more traditional explanations such as eating unripe fruits or spending too much time in the water under the sun. Traditional remedies were seen to be more effective in these cases. Households in both communities cited healers as the best treatment option for diarrhoea cases caused by heated breast milk due to a mother spending the day working under the sun, sorcery (which is marked by sudden diarrhoea, vomiting, and pain), or spirits (which may be the result of a snake looking at a mother carrying her child). Additionally, participants from both communities described seeking a healer’s help if the cause of diarrhoea were unknown or if initial treatment with traditional remedies or pharmaceuticals was unsuccessful. One herbalist described,

*Sometimes a person can confuse diarrhoea caused by heat with a diarrhoea cause by cold. That’s why one should consult a good healer. If you give the patient a plant boiled in hot water without knowing that the diarrhoea was caused by heat; then it will make the patient feel worse. That heat worsens the diarrhoea. I can tell the type of diarrhoea by measuring the person’s pulse. When it is caused by heat, the pulse is like if you had run. It’s really fast. If it is caused by cold, the pulse is really slow*. Herbalist, Community A

#### Perceived illness severity

For certain ailments, perceived severity dictated the preferred treatment. For example, certain households reported that traditional remedies were effective for diarrhoea, unless there were more than ten stools in a day, in which case pharmaceuticals were required.

#### Treatment characteristics

**Fig. 3:**
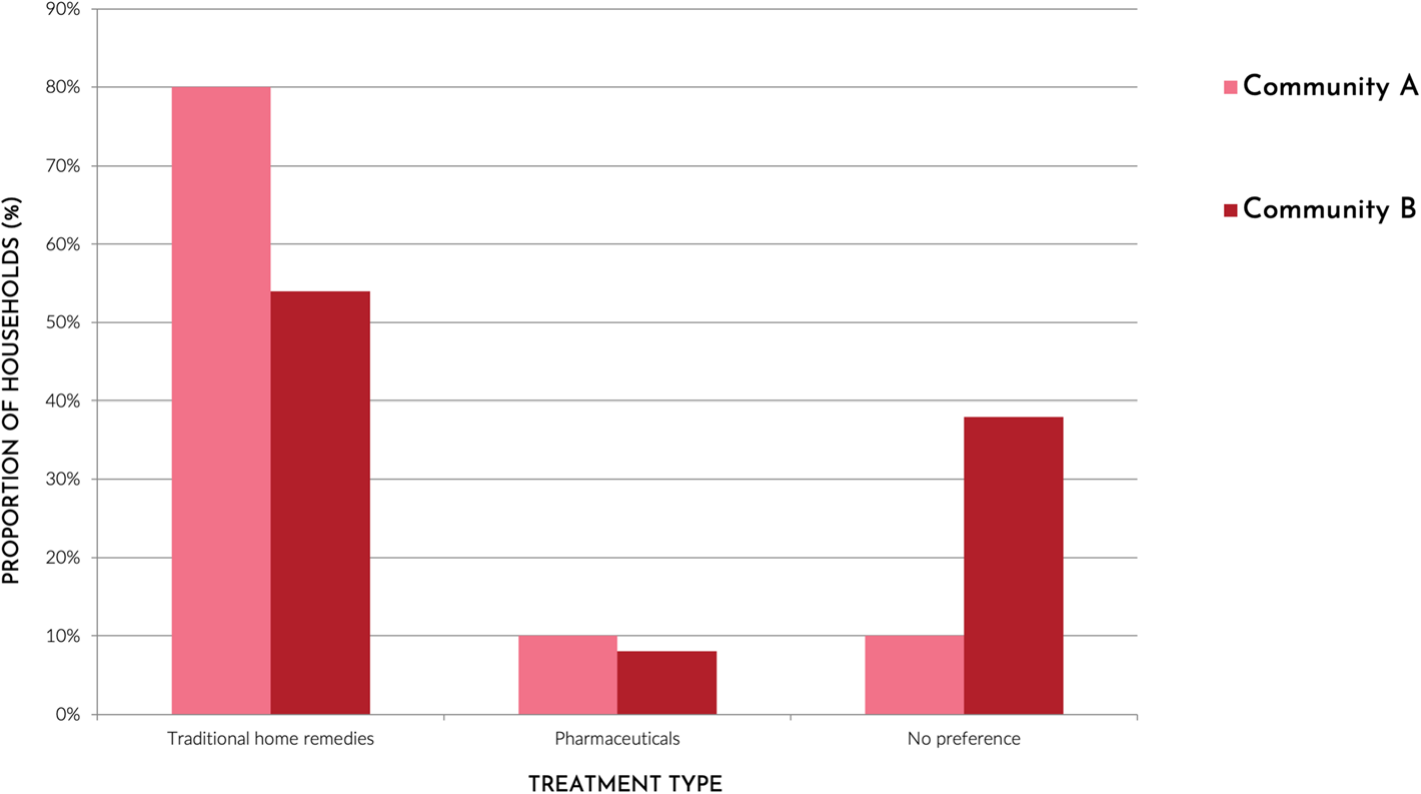
Preference for traditional home remedies compared to pharmaceuticals in two Shawi communities, Peru

Most households reported that traditional remedies were more effective than pharmaceuticals (A: 80%; B: 54%) for common ailments, which was reflected in the predominant preference for traditional remedies (Fig. 3). Widely reported reasons for this preference included that traditional medicines are fresh, natural, and treat the root cause of the disease, whereas pharmaceuticals were believed to hide pain rather than actually heal the patient. Nevertheless, due to the lengthy time required to make some traditional remedies or the occasional need to adhere to a strict diet while using these remedies, some households used pharmaceuticals as an easier alternative:

*I’ve had gastritis for 8 years. There was a time when I was feeling well. I was taking the plants that the* brujo *recommended, but I couldn’t eat meat or drink my* masato *[traditional beverage]. I couldn’t do it [follow the restrictions]. I like my wife’s* masato. Man, 40-50 years old, Community B

Similarly, households described a strong taste associated with many traditional remedies, whereas pills were *“tasteless and easier to swallow”*. Pills were, therefore, often preferred for children who may spit out traditional remedies. In contrast, several participants questioned the taste of medicinal syrups, arguing that such a sweet taste could start or worsen diarrhoea.

### Perceived healthcare barriers

During interviews, households were asked to discuss barriers to obtaining healthcare; participants focused primarily on governmental healthcare. Barriers were classified according to lack of healthcare availability, accessibility, affordability, and acceptability [11].

#### Availability

The restriction of secondary and tertiary care facilities to urban centres was a commonly cited barrier with respect to healthcare availability. Participants from both communities also reported frequent absence or shortage of health providers at the health post, which a health technician described as being partly attributable to a difficulty in retaining health personnel, since few doctors enjoy jobs in remote communities. For example, one health technician explained that,

*The obstetrician in charge only stayed here for one year. He got a better job in the city and now the post has no director. There’s only me and [a community member that cleans the post]. No one wants to work here*. Health technician

Further dissatisfaction with governmental healthcare stemmed from insufficient pharmaceutical supplies: at times, participants reported visiting the health post and being sent away due to medications being out-of-stock. Comparatively, traditional healers and herbalists were considered as being almost always available. Moreover, even in the absence of a traditional practitioner, most families could prepare basic traditional remedies, thus rendering traditional medicine more available than governmental healthcare services.

#### Accessibility

Physical access to care was a barrier for many healthcare types. Households that needed to cross the river to access the health post reported occasionally foregoing governmental healthcare, even if it was the preferred option, due to the geographical barriers. Similarly, a nurse technician described that most sick patients are unable to make the long journey to the secondary healthcare facility and are therefore referred directly from primary to tertiary care. However, travelling to Yurimaguas for tertiary care remains challenging, especially for households in Community B: “*People die here because it is too difficult to get to Yurimaguas*” (Woman, 40–50 years old, Community B). Travelling to Yurimaguas in the summer, when the river level was low, required patients to be carried on a locally constructed stretcher. This journey could take upwards of 6 h, thus increasing the risks of complications and resulting in community members dismissing this option in favour of care in the popular or traditional sectors:

“*Both plants and pharmaceuticals work well. The plants, the difference that there is [with pharmaceuticals] is that I can just take them from the* monte*, with no cost. They are more accessible. The pills … sometimes the river is too big [and I cannot get to the health centre] or the technician isn’t at the health centre.”* Man, 40-50 years old, Community B

Although fluvial transportation is possible during the winter when the river level rises, high fuel costs, low fuel availability, a lack of motorboats, and a lack of money to pay for road transportation services following the boat ride render this option impossible for most households.

It is interesting to note, however, that many participants described that some of the best and most powerful shamans are located in Chazuta district, which is a four-hour drive from Yurimaguas. Although visiting a shaman could take days of travel, participants did not cite this as an accessibility barrier.

#### Affordability

Almost all members of both communities were insured by the SIS, and could therefore receive subsidized or free governmental healthcare at a designated health facility. Nevertheless, indirect expenses, related to transportation and accommodation, barred access to higher levels of care. Households often reported foregoing necessary care to which they were referred due to an inability to pay for transportation to Yurimaguas. Similarly, a few participants who required specialized treatment in Lima reported that although most of the patient’s travel expenses are covered, no funds were provided for a travel companion. Some participants therefore sought alternative care within the community.

Similar to accessibility barriers, although participants described that the costs associated with visiting a shaman could reach between 400 and 800 *nuevos soles* (~ 120-240USD), costs of traditional healthcare were not mentioned as a barrier. Several participants even stated that they would spend all of their savings to seek a shaman’s help to treat a case of *daño* or sorcery.

#### Acceptability

Participants reported that several characteristics of governmental health facilities presented barriers with respect to healthcare acceptability. All participants noted that the staff’s inability to speak the native language affected their trust and the perceived quality of care. For example, one woman commented,

*I want a change in the health post’s personnel. They are all mestizos and when I speak in Shawi, they don’t understand me. Sometimes I cannot understand Spanish, and even if I understand I cannot speak in Spanish. I cannot trust someone I don’t understand*. Woman, 20-30 years old, Community B

In Community B, over three quarters of households also reported poor healthcare quality at the health post. Health technicians were not provided with any training regarding working in Indigenous contexts, which participants described as resulting in culturally insensitive staff. Due to these complaints of language barriers, poor treatment quality, and lack of respect, some participants reported avoiding governmental health facilities even if they were not improving with traditional remedies or knew that professional healthcare was necessary.

With respect to higher levels of care, excessive bureaucracy and lack of guidance once having reached the hospital were mentioned as barriers. Although a health technician occasionally accompanied patients to the hospital to personally refer them to a doctor, once alone, participants reported not knowing what to do. The mandatory hospital health forms were another barrier, as they are complicated and in Spanish. This was particularly problematic for women, as their understanding of Spanish was typically more limited than that of men.

## Discussion

**Fig. 4:**
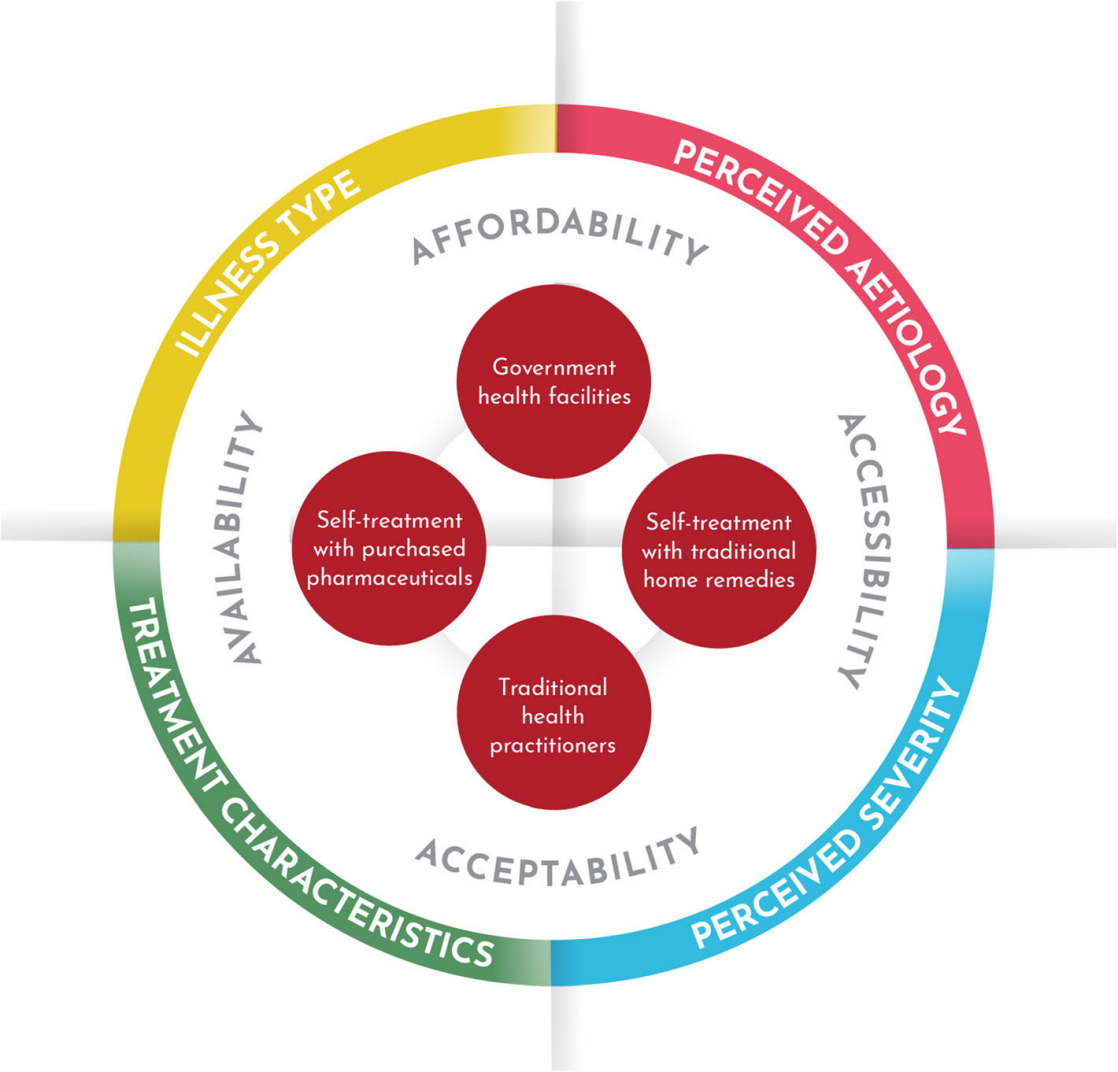
Primary health-seeking behaviours and the factors affecting their use in two Shawi communities, Peru

Consistent with other studies among Indigenous peoples in Peru [8–10, 31], Shawi participants reported plural HSBs, using a combination of treatments from all three health sectors. They held nuanced perceptions regarding the effectiveness of each healthcare option, and aimed to secure the best treatment option within their geographic, financial, and social means. Health-seeking pathways were dynamic: households navigated the plural healthcare system, constantly evaluating illness developments and considering the costs and benefits of available healthcare options [10, 12, 13, 32]. Shawi HSBs, as well as factors affecting the use of treatment options, are summarized in Fig. 4. Future studies should explore if existing explanatory models for health-seeking behaviours, such as Andersen’s Behavioural Model of Health Services, effectively characterize the health-seeking behaviours of Indigenous Peoples [33, 34].

Aligned with other studies conducted in rural settings [13, 17], Shawi women were primarily responsible for maintaining household health. Although their level of formal education was typically lower than that of men, women were responsible for recognizing illnesses and were primary contributors in HSB decision-making. Previous studies have reported that maternal, but not paternal, health knowledge in remote settings positively influences household well-being [17, 35, 36]. Consequently, public health education or interventions, aimed to promote prevention practices and encourage early treatment-seeking, might be most effective if targeted towards Shawi women [17].

Traditional health beliefs remained prevalent in both communities. Traditional home remedies were widely preferred over professional healthcare due to a combination of accessibility, affordability, and perceived effectiveness. Findings from other remote Peruvian communities are conflicting in this respect, with most studies reporting a dominant preference for traditional remedies [9, 10, 15, 31, 37, 38], whereas others report a dominant preference for professional healthcare [8, 39]. As found elsewhere [12, 14], the preference for traditional remedies was less dominant in Community B, where professional healthcare was more accessible. Nevertheless, as displayed by our findings and those from Peruvian Andean communities [10], even in communities with accessible and affordable professional healthcare, traditional treatments remained more widely preferred and accessed.

As found in other Indigenous communities in the Loreto region [8, 9] and other low-resource settings [13, 15, 17], cost and distance associated with healthcare access were among the most commonly reported barriers to accessing professional healthcare. Although the SIS scheme rendered direct medical costs affordable, as cited in the literature [11, 15], long travel distances and indirect costs associated with travel and accommodation, often barred access to professional healthcare. Nevertheless, neither cost nor distance emerged as barriers to accessing traditional healthcare. Similarly, other studies have shown that local concepts of illness and perceived treatment effectiveness can override cost and distance considerations in HSB decisions even among the poorest people [13, 39, 40].

Given the preference for traditional remedies even when professional healthcare was accessible and affordable, the perceived low quality and acceptability of current professional healthcare, and that perceived treatment effectiveness could override cost and distance barriers, simply increasing the accessibility and affordability of professional healthcare will likely be insufficient in improving health outcomes in these Shawi communities [41, 42]. Though geographic and financial constraints are indeed barriers that must be addressed, a combination of measures, including intersectoral collaboration and cultural competency training, are needed to increase healthcare acceptability and thereby improve health outcomes [41].

Despite the traditional healthcare system’s essential role in the Peruvian Amazon [2, 43], it is not recognized by, or integrated within, the official health system. In addition to being accessible and affordable, herbalists and healers were highly regarded by participants. Hence, they are an important resource and attempting to circumvent or ignore their role in healthcare provision is counter-productive and culturally insensitive [2, 36, 44].

Although no official intersectoral collaborations existed, as reported in studies from other low-resource settings [13, 32, 38], traditional practitioners reportedly referred patients to professional healthcare if the illness were beyond their scope of practice. Formalizing and improving this intercultural referral system could reduce treatment-seeking delays for diseases where professional healthcare is needed [45, 46]. Traditional practitioners could also be educated to better recognize when a referral is necessary [42]. Participants also reported that traditional practitioners would convey relevant biomedical knowledge learned from the governmental health post to their patients; health education interventions could therefore capitalize upon the community’s trust in traditional practitioners to improve health literacy and intervention effectiveness [9, 47].

Participants reported that the traditional and professional healthcare sectors were used for different disease types, and were therefore complementary rather than competitive systems [31, 45]. Similarly, professional practitioners in the Bolivian Amazon stated that while some diseases (e.g. tuberculosis) should be treated with professional healthcare, other affections (e.g. mild diarrhea) could be better treated with traditional remedies [31]. An intercultural healthcare system would allow patients to benefit from these complementary systems, and make timely and informed treatment decisions [11, 14, 31, 46, 48]. Future research should investigate viable strategies for promoting intersectoral collaboration to contribute to the development of an intercultural healthcare system [31, 48, 49].

Acceptability of professional healthcare services and providers was mentioned as an important barrier to government healthcare use. These findings aligned with several studies [14, 17, 50], where cultural differences between users and health providers led to a reduction in professional healthcare. These barriers may be partly attributable to the limited training that Peruvian medical students receive with respect to Indigenous culture and healthcare provision in Indigenous settings [43].

Neglect of local cultures and beliefs in healthcare delivery contributes to the higher disease burdens among Indigenous populations and is believed to be the biggest barrier to improving health outcomes [1, 41]. Accordingly, the World Health Organization is promoting cultural competency as a strategy to improve healthcare provision to minority groups [44]. Cultural competency is evidenced as a suitable approach to improve healthcare delivery and health outcomes amongst culturally diverse populations [40, 44, 50]. A wide variety of cultural competency interventions exist, including cultural education training, interpreters, and patient navigators [44].

As reported in other studies [37, 40], participants were reluctant to seek professional healthcare due to a general lack of respect for Indigenous cultural beliefs and traditions amongst professional healthcare providers. This reluctance could exacerbate health issues, particularly when dealing with chronic diseases that require several healthcare visits or ‘new diseases’ for which no traditional remedies exist. The success of healthcare delivery and health programs amongst Indigenous populations is contingent upon the service acknowledging and considering Indigenous culture and beliefs [2, 41]. As such, a key element to cultural competency is understanding local cultural beliefs related to health and illness such that information can be presented in a culturally appropriate and meaningful manner [41]. Professional healthcare providers should be trained to develop an appreciation of the local culture [8, 41, 44]. Furthermore, intercultural healthcare interventions, whereby interventions are adapted to align with the target population’s culture, beliefs, and behaviours are proven to increase healthcare utilization, the perceived quality of healthcare services, and user satisfaction amongst Indigenous populations [46, 48–52]. For example, the percentage of deliveries in health facilities in a Peruvian community increased from 6 to 83% through the integration of traditional Andean medical and cultural elements, such as permitting the presence of a traditional birth attendant [50]. As such, additional research is needed to better understand local cultural beliefs and needs to effectively create an intercultural healthcare system that serves Indigenous Peoples’ needs [41, 46, 50]. Furthermore, additional research that assesses the impact of racism on Shawi health-seeking behaviours would be beneficial.

## Conclusions

This study contributes new qualitative information on HSBs in remote Indigenous communities in the Peruvian Amazon. This information is prerequisite for policy-makers and stakeholders to improve health outcomes through enhanced healthcare access and use. Our findings indicate that Shawi HSBs are plural and dynamic. A variety of factors informed households’ pragmatic HSBs, including illness type, perceived aetiology, perceived severity, and treatment characteristics. Irrespective of the presence of a governmental health facility, self-treatment with traditional remedies was the primary choice for managing common illnesses. Conversely, professional healthcare was preferred for illnesses lacking traditional remedies, pregnancy check-ups, and contraceptives. Barriers impeding healthcare use included distance to healthcare facilities, healthcare costs, language barriers, and cultural insensitivity amongst professional practitioners. These barriers were considered in a complex decision-making process, and could be overridden by certain factors including perceived quality or effectiveness of care.

Concurrent strategies with respect to increasing healthcare availability, affordability, accessibility, and acceptability, are necessary to improve health outcomes in the two communities. Our findings emphasize the importance of acknowledging and considering Indigenous culture and beliefs, as well as the existing traditional medical system, within the professional healthcare system. Additional research is needed to better understand HSB decision-making in other Indigenous societies in Peru. The findings can inform regional health authorities, help strengthen the National Health Strategy of Indigenous Populations, and contribute to improving the available health services.

## Supplementary Information

**Additional file 1:.** Semi-structured interview guide used to interview participants about their health-seeking behaviours.

## Data Availability

The audio recordings and transcripts analysed in this study are not publicly available as it is not possible to sufficiently deidentify them to maintain participant privacy and confidentiality. Additional deidentified information can be obtained from the corresponding author upon reasonable request.

## Abbreviations

HSB: health-seeking behaviour
IHACC: Indigenous health adaptation to climate change
SIS: Seguro integral de salud
