# Supplementary material for: Kaniuwatewara (when we get sick): understanding health-seeking behaviours among the Shawi of the Peruvian Amazon
Source: BMC Public Health. 2021 Aug 16;21:1552. doi: 10.1186/s12889-021-11574-2 (PMC8365975; doi:10.1186/s12889-021-11574-2)
Supplement: Supplementary file 1 — Additional file 1:. Semi-structured interview guide used to interview participants about their health-seeking behaviours. [file 12889_2021_11574_MOESM1_ESM.docx]

# Interview guides

**Assessing perceptions, attitudes and practices on health and health services in four indigenous communities of the Peruvian Amazon**

**Interview guide for in depth interviews**

*Before you start, the briefing will be explained in the native language of the participant and verbal consent will be obtained. The participant can read the information document. If the participant cannot read, investigator or translator will carefully read the document. The interview will only be recorded if the participant wishes to.*

The researcher has to take note on the following before starting the interview.

|  | **Questions** | **Answers** |
| --- | --- | --- |
| **0.1** | Name of the community/sector |  |
| **0.2** | Is there a health post in the community? | Yes_ |
|  |  | No _ |
| **0.3** | Which is the closest health post? (Km/h) |  |

**Demographic questions**

|  | **Questions** | **Answers** |
| --- | --- | --- |
| **1** | Gender | F _ |
|  |  | M _ |
| **2** | How old are you? How long have you being living in the community? |  |
| **3** | Do you have a national identification document? | Yes_ N° |
|  |  | No _ |
| **4** | Do you have a health insurance (SIS / EsSalud)? | Yes_ |
|  |  | No _ |
| **5** | Are you Shipibo/Shawi? |  |
| **6** | What is your marital status? (*read options*) | Single |
|  |  | Married |
|  |  | Partner |
|  |  | Separated |
|  |  | Widow/er |
|  |  | Divorced |
| **7** | How many children do you have? (*note age and gender of each child*) |  |
| **8** | How many people live in this house? | Children |
|  |  | Adults |
| **9** | Do you know how to read and write? | Yes |
|  |  | No |
| **10** | What is the last level of studies approved? | Complete primary |
|  |  | Incomplete primary |
|  |  | Complete secondary |
|  |  | Incomplete secondary |
|  |  | Technical studies |
|  |  | University studies |
| **11** | Occupation | Agriculture |
|  |  | Artisan |
|  |  | Others |
| **12** | Partner’s occupation | Agriculture |
|  |  | Artisan |
|  |  | Others |
| **13** | How many times a year do you leave your community? |  |
| **14** | How long? How many days / weeks / months during the year are you outside your community? |  |
| **15** | In general, where do you go? |  |
| **16** | Why do you travel to those places? | It is close |
|  |  | Work |
|  |  | Health |
|  |  | Study |
|  |  | Family |
|  |  | Others |

**Health**

1. How do you define health?

*Follow-up question:* For you, what does it mean to be healthy?

2. What do you or your family need to be/feel/live healthy?

*Follow-up question:* What thing cannot miss for you or your family to be healthy?

| **To be healthy** | **It cannot be missing…** |
| --- | --- |
| Me |  |
| My family |  |
| My community |  |

3. Do you do anything special to stay healthy?

*Follow-up question:* How do you prevent illness/disease?

4. How did your grandparents prevent illness/disease?

*Follow-up question:* What did your grandparents do to be healthy?

**Illness/disease**

5. To you, what does it mean to be sick?

*Follow-up question:* What can a healthy person do, that a sick person cannot?

6. How do you know that a person is sick? How can you tell?

7. Generally, why do people get sick?

**Health care seeking**

8. How do you treat your health problems?

*Follow-up question:* What is the first thing you do when you get sick?

9. When you get sick, who do you let know first?

10. When someone in your family (adult/child/elder) gets sick what is the first thing you do?

11. Where do you go when you get sick?

12. The last time you got sick, what did you get?

*Follow-up question:* What did you do? Where did you go?

13. Once you are sick/you feel bad, how long do you wait before seeking help/care?

14. How often do you go to the post/traditional healer/other?

**Western medicine and traditional medicine**

15. In what occasions do you use the health post?

16. What diseases can only be treated in the health post?

*Follow-up question:* What diseases cannot be dealt by *curanderos*?

17. What disease can only be treated with medicinal plants/by consulting the *curandero*?

18. Do you follow the complete treatment the health technician/*curandero* gives you?

19. How long should one wait to consider a treatment is not being effective?

*Follow-up question:* How much time must pass before you decide you must change your treatment? (*Ask examples and note warning symptoms*)

20. Do you take your children to the check-ups at the health post?

*Follow-up question:* Why?

21. In your opinion, what is more effective: medicinal plants or pharmaceuticals?

*Follow-up question:* What does the effectiveness of a treatment depend on?

22. What diseases cannot be cured plants?

**Common diseases**

23. In general, what are the most common illnesses/diseases in your community?

24. What do you think is the cause of these illnesses/diseases?

25. What illnesses/diseases do men get more often?

26. What do you think causes of this health problem?

27. How do you treat this disease?

28. What illnesses/diseases do women get more often?

29. What do you think causes of this health problem?

30. How do you treat this disease?

31. What illnesses/diseases do children get more often?

32. What do you think causes of this health problem?

33. How do you treat this disease?

34. In which season (rainy/dry) do adults/children get sick more often?

35. ​​In general, are health problems increasing or decreasing in your community?

36. What is the most feared disease in the community?

*Follow-up question:* What is the most serious disease?

37. What do you do in such cases?

38. What is considered as a health emergency?

39. What do you do in such cases?

40. Are there incurable illnesses/diseases?

*Follow-up question:* Which ones?

41. What are the causes of *daño*?

*Follow-up question:* What are the symptoms?

42. Are cases of *daño* increasing/decreasing/staying the same?

*Follow-up question:* Why do you think is this the case?

**Mortality**

43. What are the causes of mortality in your community?

| **Cause** | **Very frequent** | **Not so frequent** | **Very rare** |
| --- | --- | --- | --- |
| Daño |  |  |  |
| Snake bite |  |  |  |
| … |  |  |  |

44. How long do people live in your community?

*Follow-up question:* Are there many elderly people in your community?

**Perceptions of the Ministry of Health**

45. How do the health personnel treat you when sick?

46. Do the Ministry of Health/Government conducts health programs in your community?

47. The Ministry of Health/Government conducts workshops/educational talks on health issues in your community?

48. How often do you visit a health worker (technician, nurse, etc.)? (*Only for communities without health post*)

**Access to health services**

49. Do you know the facilities and health services available to you and your family?

50. What health programs are offered at the health post?

51. How long does it take for you to get to the nearest health facility?

52. Can you go to the nearest health facility at any time of day or night?

53. Can you go to the nearest health facility during any season?

54. If you/family member are referred to the health centre/hospital, could you go there at any time of day or night?

55. If you/family member are referred to the health centre/hospital, could you go there during any season?

**Information sources**

56. Where do you get your health information?

57. Would you like to receive information about health issues in your community?

58. What do you think are the most effective ways/sources to transmit information about health issues in your community?

**Assessing knowledge, attitudes and practices on health and health services in four indigenous communities of the Peruvian Amazon**

**Interview guide for health providers**

|  | **Question** | **Answer** |
| --- | --- | --- |
| **1** | Gender | F _ |
|  |  | M _ |
| **2** | How old are you? |  |
| **3** | How long have you being working in this health facility? |  |
| **4** | What is you occupation? | Nurse technician |
|  |  | Doctos |
|  |  | Nurse |
|  |  | Others |

1. What drove you to follow this career?

2. What are your responsibilities in this position?

3. In average, how many patients that belong to Puerto Consuelo/Panaillo/Nuevo Progreso/Puerto Porvenir do you see per week?

4. How often do you visit the community? (*For technicians working in health posts outside the communities*)

**Information sources**

5. Who do you think people from the community consult when they have a question on health/health issue?

6. Do you give educational talks on relevant health issues to the community? (*Note for the researcher: It is sated among his obligations*)

*Follow-up question:* On what topics?

**Common diseases**

8. What are the most common illnesses/diseases in the community?

9. What are the most common illnesses/diseases among children in the community?

10. In your opinion, which are the most serious health problems in the community?

11. What do you think is the cause?

12. During which season (rainy/dry) do people get sicker?

*Follow-up question:* Why do you think is that?

13. How do you treat the most common diseases?

14. Do all you patients adhere to the treatments you prescribe?

15. How do you compare the treatments you give to the ones the *curanderos* give?

**Mortality**

16. What are the causes of mortality in the community?

| **Cause** | **Very frequent** | **Not so frequent** | **Very rare** |
| --- | --- | --- | --- |
| Daño |  |  |  |
| Snake bite |  |  |  |
| … |  |  |  |

**Access to health services**

17. Have you ever referred a patient to a higher level facility?

18. In what cases?

19. How do you get to the hospital?

*Follow-up question:* Does the community location/available transportation hinders or facilitates the Access to the hospital?

20. Is there anything else you would like to talk about?
